# Unsupervised learning of digit recognition using spike-timing-dependent plasticity

**DOI:** 10.3389/fncom.2015.00099

**Published:** 2015-08-03

**Authors:** Peter U. Diehl, Matthew Cook

**Affiliations:** Institute of Neuroinformatics, ETH Zurich and University ZurichZurich, Switzerland

**Keywords:** spiking neural network, STDP, unsupervised learning, classification, digit recognition

## Abstract

In order to understand how the mammalian neocortex is performing computations, two things are necessary; we need to have a good understanding of the available neuronal processing units and mechanisms, and we need to gain a better understanding of how those mechanisms are combined to build functioning systems. Therefore, in recent years there is an increasing interest in how spiking neural networks (SNN) can be used to perform complex computations or solve pattern recognition tasks. However, it remains a challenging task to design SNNs which use biologically plausible mechanisms (especially for learning new patterns), since most such SNN architectures rely on training in a rate-based network and subsequent conversion to a SNN. We present a SNN for digit recognition which is based on mechanisms with increased biological plausibility, i.e., conductance-based instead of current-based synapses, spike-timing-dependent plasticity with time-dependent weight change, lateral inhibition, and an adaptive spiking threshold. Unlike most other systems, we do not use a teaching signal and do not present any class labels to the network. Using this unsupervised learning scheme, our architecture achieves 95% accuracy on the MNIST benchmark, which is better than previous SNN implementations without supervision. The fact that we used no domain-specific knowledge points toward the general applicability of our network design. Also, the performance of our network scales well with the number of neurons used and shows similar performance for four different learning rules, indicating robustness of the full combination of mechanisms, which suggests applicability in heterogeneous biological neural networks.

## 1. Introduction

The mammalian neocortex offers an unmatched pattern recognition performance given a power consumption of only 10–20 watts (Javed et al., 2010). Therefore, it is not surprising that the currently most popular models in machine learning, artificial neural networks (ANN) or deep neural networks (Hinton and Salakhutdinov, 2006), are inspired by features found in biology. However, this comparison should be taken with a grain of salt since, despite the biological inspiration, those models use mechanisms for learning and inference which are fundamentally different from what is actually observed in biology. While ANNs rely on 32 bit or even 64 bit messages being sent between units, the neocortex uses spikes, akin to 1 bit precision (if the possible influence of spike-timing on the transmitted message is omitted). Additionally, ANN units are usually perfect integrators with a non-linearity applied after integration, which is not true for real neurons. Instead neocortical neurons are rather leaky integrators, and they use conductance-based synapses which means the change of the membrane voltage due to a spike depends on the current membrane voltage. Another non-biological aspect of ANNs is the type of learning. In ANNs the standard training method is backpropagation (Rumelhart et al., 1985), where after presenting an input example, each neuron receives its specific error signal which is used to update the weight matrix. It seems unlikely that such a neuron-specific error signal would be implemented in the brain (O'Reilly and Munakata, 2000), instead evidence is more pointing toward unsupervised learning methods like spike-timing-dependent plasticity (STDP) (Bi and Poo, 1998), which could be modulated by a global reward signal and therefore could be also used for reinforcement learning.

On the other end of the spectrum, many models in computational neuroscience are modeling biological properties very well but often they are not large scale *functional* systems. However, understanding the computational principles of the neocortex needs both aspects, the biological plausibility and good performance on pattern recognition tasks. If we only focus on biological plausibility, even if we are able to develop functional systems, it is difficult to know which mechanisms are necessary for the computation, i.e., being able to copy the system does not necessarily lead to understanding. Similarly, if we focus only on good performance we will create systems that are working well but which also do not lead to a better understanding since they are too abstract to compare them to the computational primitives of real brains. In recent years many models were developed for pattern recognition tasks that use more biologically plausible mechanisms, marrying both approaches of understanding. One popular approach is to still rely on backpropagation training but afterwards converting the ANN into a spiking neural network (SNN), which we will call “rate-based learning” (Merolla et al., 2011; O'Connor et al., 2013; Hussain et al., 2014; Neil and Liu, 2014; Diehl et al., 2015). While they show very good performance on tasks like the classical machine learning benchmark MNIST (LeCun et al., 1998), this rate-based learning is not very biologically plausible or is at least very much abstracted from the biological mechanism. Other spike-based learning methods often rely on different variants of models of STDP (Brader et al., 2007; Habenschuss et al., 2012; Beyeler et al., 2013; Querlioz et al., 2013; Zhao et al., 2014), providing a closer match to biology for the learning procedure. However, most of those models rely on a teaching signal which provides every single neuron that is used for classification with feedback indicating the correct response, which shifts the problem to the “supervisor neurons” that already need to know the solution. Also, commonly they use features in the neuron/synapse models which make learning easier but are not necessarily biologically plausible; examples include STDP models with highly application specific parameter tuning or current-based synapses, both of which often do not use graded weight changes or graded currents that are observed experimentally.

Here we present a spiking neural network which relies on a combination of biologically plausible mechanisms and which uses unsupervised learning, i.e., the weights of the network learn the structure of the input examples without using labels. It uses an architecture similar to the one presented in Querlioz et al. (2013), i.e., it uses leaky-integrate-and-fire (LIF) neurons, STDP, lateral inhibition and intrinsic plasticity. However, here we use more biologically plausible components like conductance-based synapses and different STDP rules, all with an exponential time dependence of the weight change. The possibility to vary the design of the learning rule shows the robustness of the used combination of mechanisms. We are training the network on the MNIST dataset without any preprocessing of the data (besides the necessary conversion of the intensity images to spike-trains). The performance of this approach scales well with the number of neurons in the network, and achieves an accuracy of 95% using 6400 learning neurons. Varying the learning rules but keeping the other mechanisms fixed not only shows the robustness of the framework but it also helps to better understand the relationship between the different observed mechanism types. Specifically, we observe that lateral inhibition generates competition among neurons, homoeostasis helps to give each neuron a fair chance to compete, and that in such a setup excitatory learning leads to learning prototypical inputs as receptive fields (largely independent of the learning rule used).

In the next section we explain the architecture including the neuron and synapse models, and the training and evaluation process. Section 3 contains the simulation results and in Section 4 we compare our results to those of other architectures as well as giving an intuition into how our network works.

## 2. Methods

To simulate our SNNs we used Python and the BRIAN simulator (Goodman and Brette, 2008)^1^. Here we describe the dynamics of a single neuron and a single synapse, then the network architecture and the used mechanisms, and finally we explain the MNIST training and classification procedure.

### 2.1. Neuron and synapse model

To model neuron dynamics, we chose the leaky integrate-and-fire model. The membrane voltage *V* is described by

(1) $$\begin{array}{l} \tau \frac{dV}{dt}=\left(E_{rest}-V\right)+g_{e}\left(E_{exc}-V\right)+g_{i}\left(E_{inh}-V\right) \end{array}$$

where *E*_*rest*_ is the resting membrane potential, *E*_*exc*_ and *E*_*inh*_ the equilibrium potentials of excitatory and inhibitory synapses, and *g*_*e*_ and *g*_*i*_ the conductances of excitatory and inhibitory synapses, respectively. As observed in biology, we use a time constant τ, which is longer for excitatory neurons than for inhibitory neurons. When the neuron's membrane potential crosses its membrane threshold *v*_*thres*_, the neuron fires and its membrane potential is reset to *v*_*reset*_. Within the next few milliseconds after the reset, the neuron is in its refractory period and cannot spike again.

Synapses are modeled by conductance changes, i.e., synapses increase their conductance instantaneously by the synaptic weight *w* when a presynaptic spike arrives at the synapse, otherwise the conductance is decaying exponentially. If the presynaptic neuron is excitatory, the dynamics of the conductance *g*_*e*_ are

(2) $$\begin{array}{l} \tau_{g_{e}}\frac{dg_{e}}{dt}=-g_{e} \end{array}$$

where τ_*g*_*e*__ is the time constant of an excitatory postsynaptic potential. Similarly, if the presynaptic neuron is inhibitory, a conductance *g*_*i*_ is updated using the same equation but with the time constant of the inhibitory postsynaptic potential τ_*g*_*i*__.

We use biologically plausible ranges for almost all of the parameters in our simulations, including time constants of membranes, synapses and learning windows (Jug, 2012); the exception is the time constant of the membrane voltage of excitatory neurons. Increasing the time constant of the excitatory neuron membrane potential to 100 ms (from 10 to 20 ms that are typically observed for biological neurons), greatly increased the classification accuracy. The reason is that rate-coding is used to represent the input, see Section 2.5, and therefore longer neuron membrane constants allow for better estimation of the input spiking rate. For example, if the recognition neuron can only integrate inputs over 20 ms at a maximum input rate of 63.75 Hz, the neuron will only integrate over 1.275 spikes on average, which means that a single noise spike would have a large influence. By increasing the membrane time constant to 100 ms, a neuron can integrate over 6.375 spikes on average, reducing the effects of noise. The problem of too few inputs spikes only exists since the architecture uses a much lower number of input neurons than biologically observed to increase simulation speed. An increase of the number of input neurons would allow for the same averaging effect.

### 2.2. Network architecture

**Figure 1:**
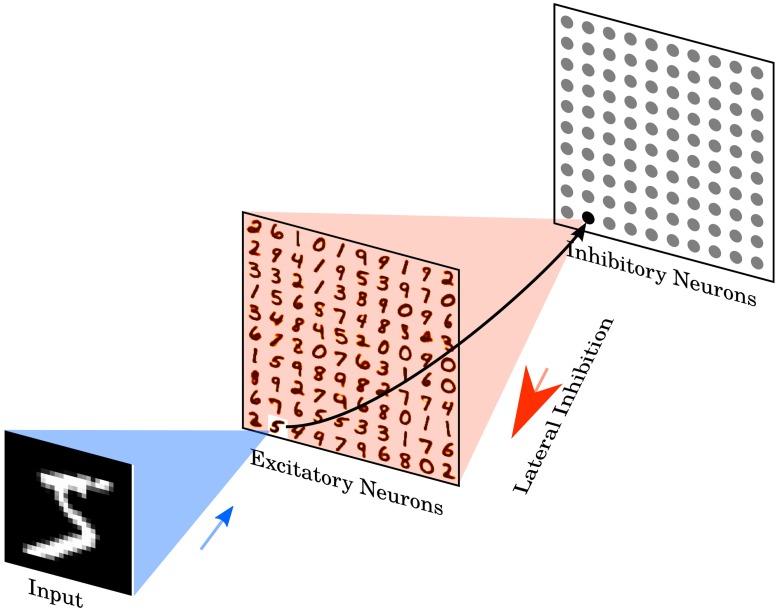
**Network architecture**. The intensity values of the 28 × 28 pixel MNIST image are converted to Poisson-spike with firing rates proportional to the intensity of the corresponding pixel. Those Poisson-spike trains are fed as input to excitatory neurons in an all-to-all fashion. The blue shaded area shows the input connections to one specific excitatory example neuron. Excitatory neurons are connected to inhibitory neurons via one-to-one connections, as shown for the example neuron. The red shaded area denotes all connections from one inhibitory neuron to the excitatory neurons. Each inhibitory neuron is connected to all excitatory neurons, except for the one it receives a connection from. Class labels are not presented to the network, so the learning is unsupervised. Excitatory neurons are assigned to classes after training, based on their highest average response to a digit class over the training set. No additional parameters are used to predict the class, specifically no linear classifier or similar methods are on top of the SNN.

The network consists of two layers, see Figure 1. The first layer is the input layer, containing 28 × 28 neurons (one neuron per image pixel), and the second layer is the processing layer, containing a variable number of excitatory neurons and as many inhibitory neurons. Each input is a Poisson spike-train, which is fed to the excitatory neurons of the second layer. The rates of each neuron are proportional to the intensity of the corresponding pixel in the example image, see Section 2.5.

The excitatory neurons of the second layer are connected in a one-to-one fashion to inhibitory neurons, i.e., each spike in an excitatory neuron will trigger a spike in its corresponding inhibitory neuron. Each of the inhibitory neurons is connected to all excitatory ones, except for the one from which it receives a connection. This connectivity provides lateral inhibition and leads to competition among excitatory neurons. The maximum conductance of an inhibitory to excitatory synapse is fixed at 10 nS. However, the exact value did not have a big influence on the results of the simulation, instead the ratio between inhibitory and excitatory synaptic conductance has to be balanced to ensure that lateral inhibition is neither too weak, which would mean that it does not have any influence, nor too strong, which would mean that once a winner was chosen that winner prevents other neurons from firing.

### 2.3. Learning

All synapses from input neurons to excitatory neurons are learned using STDP. To improve simulation speed, the weight dynamics are computed using synaptic traces (Morrison et al., 2007). This means that, besides the synaptic weight, each synapse keeps track of another value, namely the presynaptic trace *x*_*pre*_, which models the recent presynaptic spike history. Every time a presynaptic spike arrives at the synapse, the trace is increased by 1, otherweise *x*_*pre*_ decays exponentially. When a postsynaptic spike arrives at the synapse the weight change Δ*w* is calculated based on the presynaptic trace

(3) $$\begin{array}{l} \Delta w=\eta \left(x_{pre}-x_{tar}\right){\left(w_{max}-w\right)}^{\mu } \end{array}$$

where η is the learning-rate, *w*_*max*_ is the maximum weight, and μ determines the dependence of the update on the previous weight. *x*_*tar*_ is the target value of the presynaptic trace at the moment of a postsynaptic spike. The higher the target value, the lower the synaptic weight will be. This offset ensures that presynaptic neurons that rarely lead to firing of the postsynaptic neuron will become more and more disconnected and is especially useful if the postsynaptic neuron is only rarely active. A similar effect can be achieved by adding some noise to the input and adding a weight decrease mechanism to the learning rule (like in classical STDP, Bi and Poo, 1998) to disconnect irrelevant inputs. However, in our simulations it comes at the cost of an increased simulation time. The learning rule is similar to the one used in Querlioz et al. (2013) but here we use an exponential time dependence which is more biologically plausible (Abbott and Song, 1999) than a time independent weight change.

In order to compare the robustness of the chosen architecture to the exact form of the learning rule, we tested three other STDP learning rules. The second STDP rule uses an exponential weight dependence (Nessler et al., 2013; Querlioz et al., 2013) to compute the weight change

(4) $$\begin{array}{l} \Delta w=\eta_{post}\left(x_{pre}\exp\left(-\beta w\right)-x_{tar}\exp\left(-\beta \left(w_{max}-w\right)\right)\right) \end{array}$$

where β determines the strength of the weight dependence.

The third rule uses not only a presynaptic trace but also a postsynaptic trace, which works in the same way as the presynaptic trace but its increase is triggered by a postsynaptic spike. Additionally, for this learning rule weight changes occur for pre- and postsynaptic spikes. The weight change Δ*w* for a presynaptic spike is

(5) $$\begin{array}{l} \Delta w=-\eta_{pre}x_{post}w^{\mu } \end{array}$$

where η_*pre*_ is the learning-rate for a presynaptic spike and μ determines the weight dependence. The weight change for a postsynaptic spike is

(6) $$\begin{array}{l} \Delta w=\eta_{post}\left(x_{pre}-x_{tar}\right){\left(w_{max}-w\right)}^{\mu } \end{array}$$

where η_*post*_ is the learning rate, *w*_*max*_ is the maximum weight, and *x*_*tar*_ is the target average value of the presynaptic trace at the moment of a postsynaptic spike.

Additionally, we learned the weights of the network using the triplet STDP rule (Pfister and Gerstner, 2006). Since this rule does not use any weight dependence for learning, we either need to incorporate it in the rule or we need to restrict the weights in some other form. Here we use divisive weight normalization (Goodhill and Barrow, 1994), which ensures an equal use of the neurons.

Note that the power-law and the exponential weight-dependence STDP rule have the advantage that weight updates are triggered only when a spike is fired by a postsynaptic excitatory neuron. Since the firing rate of the postsynaptic neurons is quite low, a more complex STDP update for postsynaptic firing doesn't require many computational resources. The symmetric learning rule and the triplet rule are computationally more expensive to simulate using software simulations (especially for larger networks) since for every presynaptic event the weight change has to be calculated for every single postsynaptic neuron.

### 2.4. Homoeostasis

The inhomogeneity of the input leads to different firing rates of the excitatory neurons, and lateral inhibition further increases this difference. However, it is desirable that all neurons have approximately equal firing rates to prevent single neurons from dominating the response pattern and to ensure that the receptive fields of the neurons differentiate. To achieve this, we employ an adaptive membrane threshold resembling intrinsic plasticity (Zhang and Linden, 2003). Specifically, each excitatory neuron's membrane threshold is not only determined by *v*_*thresh*_ but by the sum *v*_*thresh*_ + θ, where θ is increased every time the neuron fires and is exponentially decaying (Querlioz et al., 2013). Therefore, the more a neuron fires, the higher will be its membrane threshold and in turn the neuron requires more input to spike in the near future. Using this mechanism, the firing rate of the neurons is limited because the conductance-based synapse model limits the maximum membrane potential to the excitatory reversal potential *E*_*exc*_, i.e., once the neuron membrane threshold is close to *E*_*exc*_ (or higher) it will fire less often (or even stop firing completely) until θ decreases sufficiently.

### 2.5. Input encoding

The input to the network is based on the MNIST dataset which contains 60,000 training examples and 10,000 test examples of 28 × 28 pixel images of the digits 0–9 (LeCun et al., 1998). The input is presented to the network for 350 ms in the form of Poisson-distributed spike trains, with firing rates proportional to the intensity of the pixels of the MNIST images. Specifically, the maximum pixel intensity of 255 is divided by 4, resulting in input firing rates between 0 and 63.75 Hz. Additionally, if the excitatory neurons in the second layer fire less than five spikes within 350 ms, the maximum input firing rate is increased by 32 Hz and the example is presented again for 350 ms. This process is repeated until at least five spikes have been fired during the entire time the particular example was presented.

### 2.6. Training and classification

To train the network, we present digits from the MNIST training set (60,000 examples) to the network. Before presenting a new image, there is a 150 ms phase without any input to allow all variables of all neurons decay to their resting values (except for the adaptive threshold). After training is done, we set the learning rate to zero, fix each neuron's spiking threshold, and assign a class to each neuron, based on its highest response to the ten classes of digits over one presentation of the training set. This is the only step where labels are used, i.e., for the training of the synaptic weights we do not use labels.

The response of the class-assigned neurons is then used to measure the classification accuracy of the network on the MNIST test set (10,000 examples). The predicted digit is determined by averaging the responses of each neuron per class and then choosing the class with the highest average firing rate.

## 3. Results

**Figure 2:**
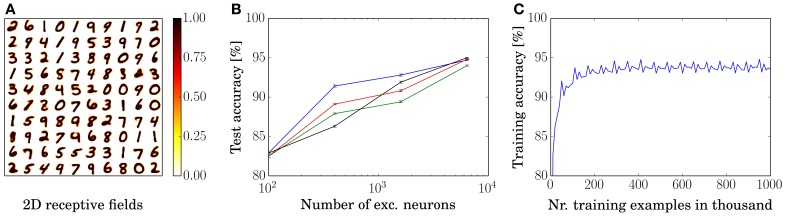
**Training results. (A)** Rearranged weights (from 784 to 28 × 28) of the connections from input to excitatory neurons of for a network with 100 excitatory neurons in a 10 by 10 grid. **(B)** Performance as a function of the number of excitatory neurons. Each dot shows the performance for a certain network size as an average over ten presentations of the entire MNIST test set, during which no learning occurs. Error bars denote the standard deviation between ten presentations of the test set. Performances of each of the learning rules are denoted by black (power-law weight dependence STDP), red (exponential weight dependence STDP), green (pre-and-post STDP), and blue lines (triplet STDP), respectively. **(C)** Training accuracy as a function of presented training examples. The last 10,000 digits are used for assigning labels to the neurons for the current 10,000 digits, e.g., examples 30,001–40,000 are used to assign the labels to classify for examples 40,001–50,000. Shown is the graph for the 1600 excitatory neuron network with symmetric learning rule.

We trained and tested a network with 100 excitatory neurons by presenting 40,000 examples of the MNIST training set. The resulting rearranged input to excitatory neuron weights are shown in Figure 2A. For each neuron, the 784-dimensional input vector is rearranged into a 28 × 28 matrix to visualize that the neurons learn prototypical inputs.

Additionally to the 100 neuron network, we trained and tested three other networks with 400, 1600, and 6400 excitatory neurons by presenting 3, 7, and 15 times the entire MNIST training set; the four networks achieved an average classification accuracy of 82.9, 87.0, 91.9, and 95.0% for the power-law weight dependence STDP rule, respectively. For all simulations, we used the same neuron, synapse, and STDP parameters (except for the parameters of the adaptive threshold and the inhibition strength which needed to be adapted to keep a constant response rate). The accuracies are averaged over ten presentations of the 10,000 examples of the MNIST test set, see Figure 2B. Since the intensity images of the MNIST test set are converted into Poisson-distributed spike trains, the accuracy can differ for different spike timings. However, the standard deviation of the performance over ten presentations of the entire test set (using the same trained network) is small (≈0.1%), as shown in the error bars in Figure 2B.

The performance for the remaining three learning rules are also depicted in Figure 2B, where the exponential weight dependence is shown in red, the performance using pre-and-post STDP is shown in green, and the performance using the triplet STDP rule is shown in blue. Training accuracy of the 1600 neuron network with a symmetric rule is shown in Figure 2C. After approximately 200,000 examples the performance is close to its convergence and even after one million examples performance does not go down but stays stable. Note that the periodic structure is due to the repeated presentation of the MNIST training set. This trend is the same for all network sizes and learning rules. However, bigger networks need longer to train until they reach peak performance.

**Figure 3:**
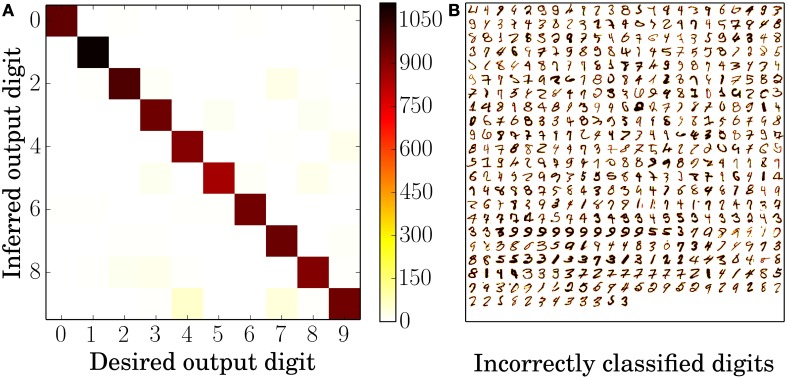
**Error analysis**. **(A)** Average confusion matrix of the testing results over ten presentations of the 10,000 MNIST test set digits. High values along the identity indicate correct identification whereas high values anywhere else indicate confusion between two digits, for example the digits 4 and 9. **(B)** All 495 incorrectly classified digits of one classification run over all 10,000 MNIST test set digits. The darker a pixel of the digit, the higher is its intensity value and therefore the frequency of input spikes. Both plots are based on the 6400 excitatory neuron network with the standard STDP rule.

An error analysis for the 6400 neuron network using the standard STDP rule is depicted in Figure 3. Figure 3A shows the average confusion matrix over ten presentations of the MNIST test set, i.e., every single classification of the test examples belongs to one of the 10 by 10 tiles and its position is determined by the actual digit and the inferred digit. Not surprisingly, given a classification rate of 95%, most examples are on the identity which corresponds to correct classification; more interesting are the misclassified examples. The most common confusions are that 4 is 57 times identified as 9, 7 is identified ≈40 times as 9 and 7 is ≈26 times identified as 2. While 4 and 9, and 7 and 2 are easily confused it does not seem immediately obvious that a 7 could be mistaken as a 9. The likely explanation can be seen in Figure 3B. Often the only distinguishing feature between the misclassified 7's and a typical 9 is that the middle horizontal stroke in the 7 is not connected to upper stroke, which means that neurons which have a receptive field of a 9 are somewhat likely to fire as well.

Since each neuron only responds to a very small subset of input digits, the responses are very sparse and only very few spikes are fired per example. Even in the biggest network with 6400 excitatory neurons, only ≈17 spikes are fired in response to one digit presentation. Specifically, for correctly identified examples, ≈16 spikes are fired from neurons from the same class and ≈1 spike from neurons assigned to a different class, whereas for incorrectly identified examples ≈3 spikes were fired from neurons of the correct class and ≈12 spikes from neurons of the other classes.

## 4. Discussion

### 4.1. Comparison

The presented network achieves good classification performance on the MNIST benchmark using SNNs with unsupervised learning made of biologically plausible components. A comparison of spiking neural networks used for MNIST classification is shown in Table 1. On the 10,000 digit test set, a difference of 0.1% is statistically significant (Larochelle et al., 2009). Note that Neftci et al. (2013) and Hussain et al. (2014) tested their networks only on 1000 and 5000 digits, respectively. Also, Brader et al. (2007) used 10,000 digits for testing which were randomly drawn from the MNIST dataset instead of the dedicated MNIST test set, which tests for memorization rather than generalization. One common way the weights are trained is using a rate-based algorithm and then transferring those trained weights into a SNN (referred to as “Rate-based” training in Table 1). Typically, the training procedure used for such rate-based training is based on popular models in machine learning like the Restricted Boltzman Machine (RBM) or convolutional neural networks. The best performance on the MNIST benchmark achieved using this conversion method is 99.1% (Diehl et al., 2015). Another approach is to train the weights using spike-based training procedures, typically relying on STDP in combination with a teaching signal. Using our unsupervised training method we were able to achieve up to 95% classification accuracy, which is clearly less than than the best spiking networks with less biologically plausible methods but given the very simple design of the network there is room for improvement, for example by localizing receptive fields and increasing the number of layers to learn more abstract features.

**Table 1:** **Classification accuracy of spiking neural networks on MNIST test set**.

**Architecture**	**Preprocessing**	**Training-type**	**(Un-)supervised**	**Learning-rule**	**Performance**
Dendritic neurons (Hussain et al., 2014)	Thresholding	Rate-based	Supervised	Morphology learning	90.3%
Spiking RBM (Merolla et al., 2011)	None	Rate-based	Supervised	Contrastive divergence, linear classifier	89.0%
Spiking RBM (O'Connor et al., 2013)	Enhanced training set to 120,000 examples	Rate-based	Supervised	Contrastive divergence	94.1%
Spiking convolutional neural network (Diehl et al., 2015)	None	Rate-based	Supervised	Backpropagation	99.1%
Spiking RBM (Neftci et al., 2013)	Thresholding	Rate-based	Supervised	Contrastive divergence	92.6%
Spiking RBM (Neftci et al., 2013)	Thresholding	Spike-based	Supervised	Contrastive divergence	91.9%
Spiking convolutional neural network (Zhao et al., 2014)	Scaling, orientation detection, thresholding	Spike-based	Supervised	Tempotron rule	91.3%
Two layer network (Brader et al., 2007)	Edge-detection	Spike-based	Supervised	STDP with calcium variable	96.5%
Multi-layer hierarchical network (Beyeler et al., 2013)	Orientation-detection	Spike-based	Supervised	STDP with calcium variable	91.6%
Two layer network (Querlioz et al., 2013)	None	Spike-based	Unsupervised	Rectangular STDP	93.5%
Two layer network (this paper)	None	Spike-based	Unsupervised	Exponential STDP	95.0%

A network architecture similar to ours is presented in Querlioz et al. (2011a, 2013) and Bichler et al. (2012), using the learning rule presented in Querlioz et al. (2011b). The main differences between their network and ours is that we show the robustness to different learning rules and we use more biologically plausible mechanisms, which include exponential conductance-based synapses instead of a current-based synapses, exponential shaped STDP time-windows instead of a rectangular ones, and inhibition is applied using an inhibitory exponential conductance instead of clamping the postsynaptic membrane voltage to the reset value for a predefined inhibition time. Especially the latter modification makes learning more difficult, i.e., using 400 excitatory neurons for each one of the networks, the one in Querlioz et al. (2013) outperforms the one presented here with the same size by about 2%. The likely reason for the increased difficulty in learning is that in Querlioz et al. (2013) learning works best when *t*_*inh*_ is equal to the refractory period of the neuron, such that after one neuron fires, all neurons have the same chance of firing after the refractory period. It is not easily possible to achieve the same effect using inhibitory exponential conductances, since it would be necessary to simultaneously fine tune the time constant of the inhibitory conductance, the refractory period, and the strength of the connection from inhibitory to excitatory neurons. Even if such a fine tuning is achieved, neurons that are not in their refractory period can still integrate incoming excitatory potentials and thus increase their chance of firing.

Another approach to unsupervised learning with spiking neural networks is presented in Masquelier and Thorpe (2007) and Kheradpisheh et al. (2015), where they use temporal spike-coding in combination with a feature hierarchy to achieve impressive results on different vision tasks and even outperforming deep convolutional networks in 3D object recognition.

### 4.2. Inhibition

In the current implementation we used as many inhibitory neurons as excitatory neurons, such that every spike of an excitatory neuron (indirectly) leads to an inhibition of all other excitatory neurons. We chose this more direct implementation of a soft winner-take-all mechanism to reduce computational complexity. This can be changed to a more biologically plausible architecture by substituting the big pool of inhibitory neurons with a smaller one to match the biologically observed 4:1 ratio of excitatory to inhibitory neurons, and by using a one-to-many connectivity from excitatory to inhibitory neurons. This would result in a network where a spike of an excitatory neuron leads to inhomogeneous inhibitory inputs to other excitatory neurons and thus might favor the activation of some neurons over others. Nonetheless, the adaptive threshold might counterbalance some of the effect and also in a big network those effects should be averaged out, which means that the performance of the network should stay approximately the same.

### 4.3. Spike-based learning for machine learning

Since energy consumption is a major cost factor for companies with lots of data (Barroso, 2005), there is a strong motivation to decrease power consumption of chips. Current implementations of spiking neural networks (SNN) on neuromorphic hardware (Indiveri et al., 2006; Khan et al., 2008; Benjamin et al., 2014; Merolla et al., 2014) use only a few nJ or even pJ for transmitting a spike (Merolla et al., 2011; Park et al., 2014; Mayr et al., 2015) (for some setups as little energy as 0.02 pJ per spike, Azghadi et al., 2014) and consume only few pW of power per synapse (Rahimi Azghadi et al., 2014); some of those neuromorphic systems also offer on-chip learning mechanisms (Indiveri et al., 2006; Diehl and Cook, 2014; Galluppi et al., 2014).

Given that power consumption is most likely going to be one of the main reasons to use neuromorphic hardware in combination with spike-based machine learning architectures, it may be preferable to use spike-based learning instead of rate-based learning since the learning procedure itself has a high power consumption (note however that both methods are spike-based during test time). Specifically, spike-based learning is important when the learning procedure takes up a significant part of time the network will be used. Another application where spike-based learning is needed is for systems which have to adapt dynamically to their environment, i.e., when it's not enough to train the system once and run it with the pre-trained weights. Possible examples include speech recognition systems that are pre-trained but adaptable to the user's accent, or vision processors that have to be tuned to the specific vision sensor. Such an adaptive vision processing system is especially interesting in conjunction with a spiking vision sensor like the ATIS or the DVS (Lichtsteiner et al., 2008; Leñero-Bardallo et al., 2010; Posch et al., 2010) as it provides an end-to-end low-power spike-based vision system. If our network were implemented on a low-power neuromorphic chip (Indiveri et al., 2006; Khan et al., 2008; Benjamin et al., 2014; Merolla et al., 2014), it could be run on a very low power budget; for example, using IBM's TrueNorth chip (Merolla et al., 2014) which consumes about 72 mW for 1 million neurons, the network would consume less than 1 mW.

### 4.4. Competitive learning

Intuitively, the function of the network is similar to competitive learning procedures (McClelland et al., 1986) like self-organizing maps (Kohonen, 1990) or neural-gas (Fritzke, 1995), which share aspects with k-means. This analogy to k-means-like learning algorithms is especially interesting since recently such approaches have been shown to be very successful in complex machine learning tasks (Coates and Ng, 2012). An in-depth analysis of expectation-maximization in a spiking network can be found in Habenschuss et al. (2012). The main idea is that each neuron learns and represents one prototypical input or an average of some similar inputs. Every time an input is presented, the network determines the prototypes that are most similar to the particular input. Those winner prototypes are then used to predict the class of the input and their weights are adapted such that they become more similar to the current input. In our network this means that every time a neuron spikes, because an example is similar enough to its receptive field, it will make its receptive field more similar to the example. The lateral inhibition prevents the prototypes from becoming too similar to each other (which means that they spread out in the input space), since only a few different neurons will be able to respond to each example and in turn only a few neurons can adapt their receptive fields toward it. Homoeostasis can be thought of as a tool to keep an approximately constant number of examples within range of the prototype. In the extreme of presenting as many different input examples as neurons learning them, the network is more similar to k-nearest-neighbors (kNN). This hints that the peak performance of the architecture presented here by just increasing the number of neurons is probably around 95–97% as it is for kNN methods without preprocessing (LeCun et al., 1998). However, it is probably possible to increase the performance further by using more layers of the same architecture as was done in Coates and Ng (2012).

### 4.5. Robustness of learning

We showed that using four different STDP rules together with lateral inhibition and homoeostasis, the resulting networks have a similar performance and show very stable learning over time. Especially the latter property is commonly hard to achieve since many networks tend to overfit the data, or lack mechanisms to prevent weights from growing too much. Figure 2C shows that the network already performs well after presenting 60,000 examples but also that it does not show a decrease in performance even after one million examples. The reason why the performance of the network is so stable over time is most likely the competition between neurons, which forces the neurons to learn as different input patterns as possible, and the weight dependence of the learning, which prevents weights from growing big if the input patterns do not reflect it. This flexibility in regards to number of learning examples and changes of the implementation is crucial in real biological systems, where we find very heterogeneous cells for different animals of the same species and even different properties in the same animal for different cells.

The other advantages of our system are its scalability, enabling a trade-off between computational cost and performance, and its flexibility in spike-based unsupervised learning rules, allowing training the network without labels and using only a few labels to assign neurons to classes.

### Conflict of interest statement

The authors declare that the research was conducted in the absence of any commercial or financial relationships that could be construed as a potential conflict of interest.

## Footnotes

^1^The code, including used parameters, is available under https://github.com/peter-u-diehl/stdp-mnist (only the Brian package has to be installed additionally to the standard python packages to run the code).
